# Semi-LASER localized dynamic ^31^P magnetic resonance spectroscopy in exercising muscle at ultra-high magnetic field

**DOI:** 10.1002/mrm.22730

**Published:** 2011-03-07

**Authors:** Martin Meyerspeer, Tom Scheenen, Albrecht Ingo Schmid, Thomas Mandl, Ewald Unger, Ewald Moser

**Affiliations:** 1 Center for Medical Physics and Biomedical Engineering, Medical University of ViennaWien, Austria; 2 MR Centre of Excellence, Medical University of ViennaWien, Austria; 3 Department of Radiology, Radboud University Nijmegen Medical CentreNijmegen, The Netherlands; 4 Department of Clinical Pharmacology, Medical University of ViennaWien, Austria

**Keywords:** dynamic MRS, exercising muscle, phosphorous, localized MRS, high field

## Abstract

Magnetic resonance spectroscopy (MRS) can benefit from increased signal-to-noise ratio (SNR) of high magnetic fields. In this work, the SNR gain of dynamic ^31^P MRS at 7 T was invested in temporal and spatial resolution. Using conventional slice selective excitation combined with localization by adiabatic selective refocusing (semi-LASER) with short echo time (TE = 23 ms), phosphocreatine quantification in a 38 mL voxel inside a single exercising muscle becomes possible from single acquisitions, with SNR = 42 ± 4 in resting human medial gastrocnemius.

The method was used to quantify the phosphocreatine time course during 5 min of plantar flexion exercise and recovery with a temporal resolution of 6 s (the chosen repetition time for moderate *T*_1_ saturation). Quantification of inorganic phosphate and pH required accumulation of consecutively acquired spectra when (resting) Pi concentrations were low. The localization performance was excellent while keeping the chemical shift displacement acceptably small. The SNR and spectral line widths with and without localization were compared between 3 T and 7 T systems in phantoms and in vivo.

The results demonstrate that increased sensitivity of ultra-high field can be used to dynamically acquire metabolic information from a clearly defined region in a single exercising muscle while reaching a temporal resolution previously available with MRS in non-localizing studies only. The method may improve the interpretation of dynamic muscle MRS data. Magn Reson Med, 2011. © 2011 Wiley-Liss, Inc.

Magnetic resonance spectroscopy (MRS) has been used for studying the metabolic response of muscle tissue to exercise for decades (1). Particularly, ^31^P MRS has a long tradition for measuring concentrations, as ratios or in absolute millimolar quantities, and rate constants of high-energy phosphates in muscle (2–7), as well as in liver (8–10) and brain (11, 12). In many dynamic ^31^P MRS studies, the signal is not localized beyond the volume selection resulting from choosing a ratio frequency (RF) transmit/receive coil of appropriate size, typically about 10 cm in diameter, for application in humans. This is mainly due to the lower signal to noise ratio (SNR) compared to proton MRS of the same tissue obtained in comparable measurement time, as well as due to the simplicity and robustness that pulse-acquire MRS offers to clinical research.

Nevertheless, several factors motivate the acquisition of localized, dynamic ^31^P spectroscopy: exercise may be distributed heterogeneously across a volume containing different muscle groups, localization to focal lesions may be of particular interest, and when comparing ^31^P MRS data to localization specific data (e.g., biopsies or single-voxel ^1^H MRS data), drawing conclusions based on the comparison with non-localized data may be misleading, as data are representative for different tissue types and volumes. Finally, because of the precise definition of the volume of interest (VOI), localized MRS opens the possibility of accurate absolute quantification via the phantom replacement technique (13).

The increased specificity of localized MRS comes at the cost of lower SNR per unit time, which may in turn necessitate temporal averaging, thus resulting in low temporal resolution. This potentially increases motion-related artifacts and renders its application to clinical trials less probable or successful. A key feature of a dynamic measurement is its capability to deliver reliable data with a temporal resolution higher than the typical changes to be observed. The dynamics in MR signals during exercise and recovery occur with half times on the order of minutes and below. To resolve these time courses with several appropriately spaced data points and, consequently, fit a numerical model of monoexponential kinetics, temporal resolutions on the order of seconds are required. With the introduction of ultra-high field whole-body MR scanners (7 T and beyond) for research application (14), the gap between high temporal resolution, non-localized ^31^P MRS, and dynamic, localized MRS has narrowed. The purpose of this work is to examine the applicability of dynamic ^31^P MRS to one particular muscle during exercise, using gradient-based single voxel localization employing a conventional slice selective excitation combined with localization by adiabatic selective refocusing (semi-LASER), (15–17). The adiabatic refocusing RF pulses used in the semi-LASER sequence have the advantages of high bandwidth (BW) and of alleviating the effects of the inhomogeneous amplitude of the radio frequency (*B*_1_) field of a simple loop coil that was used for excitation and signal detection. The pulse sequence was implemented on a 7 T whole-body system. By exploiting the gain in SNR achievable at 7 T in combination with this acquisition method, the goal was to increase specificity of dynamic ^31^P MRS in terms of selectively acquiring signal from a single muscle, without the need to reduce temporal resolution below the limit of a single acquisition, as is a common practice with unlocalized pulse-acquire ^31^P MRS at lower fields (4, 7, 18). There are several challenges connected with dynamic MRS at ultra-high magnetic field (*B*_0_). Susceptibility artifacts scale proportionally with *B*_0_ which potentially annihilate the SNR benefit, particularly in the presence of motion in an exercise study. Because of larger spectral dispersion, the necessary excitation bandwidth increases with field strength, which, in a conventional approach, necessitates shorter RF pulses with higher *B*_1_ amplitudes. However, the applicable RF power is restricted due to technical limitations of the coil as well as due to specific absorption rate (SAR) limits.

Here, we demonstrate that with the chosen excitation scheme, localized dynamic ^31^P MRS has strong potential to become a useful tool in physiological and clinical studies of human skeletal muscle function and metabolism.

## SUBJECTS AND METHODS

Healthy subjects (*n* = 8, two females, aged 30 ± 8 years, body mass index = 24 ± 4) performed plantar flexion exercise in supine position on a custom-built ergometer with a pneumatic system generating the force (as described in Ref.^19^ and a similar version with improved adaption to the particular MR scanner's patient bed). Written informed consent was obtained prior to the examinations in accordance to the regulations of the local ethics committee. The subjects were instructed to perform two plantar flexions per repetition time (TR) of the sequence (i.e., 6 s) over the normal range of foot flexion in distal direction. To minimize motion-related artifacts (i.e., localization of different fractions of muscle tissue, potentially also causing line broadening and effects of altered coil load), the subjects were trained to return their foot to the neutral position before each MR excitation and acquisition, which was triggered acoustically by gradient noise. The pedal force was adjusted via the pressure in the ergometer's pneumatic system to achieve submaximal exercise and to yield significant phosphocreatine (PCr) depletion (see Results section).

For RF transmission and reception of NMR signals, a dual-tuned transmit-receive loop coil was used. The coil with a diameter of 10.5 cm for ^31^P and 9.5 cm for ^1^H (Rapid Biomedical, Würzburg, Germany) was interfaced to a Siemens 7 T whole-body MR system (Siemens Medical Solutions, Erlangen, Germany). The manufacturer's implementation of a 3D map shim was used for localized first- and second- order shimming in the VOI. Calibration of RF transmit voltage for achieving 90° excitation tip angles and fulfilling adiabatic conditions was verified by varying the RF transmit voltage until a maximum of the PCr signal was reached for the given geometry, individually for each subject, prior to the dynamic measurements.

**Fig. 1:**
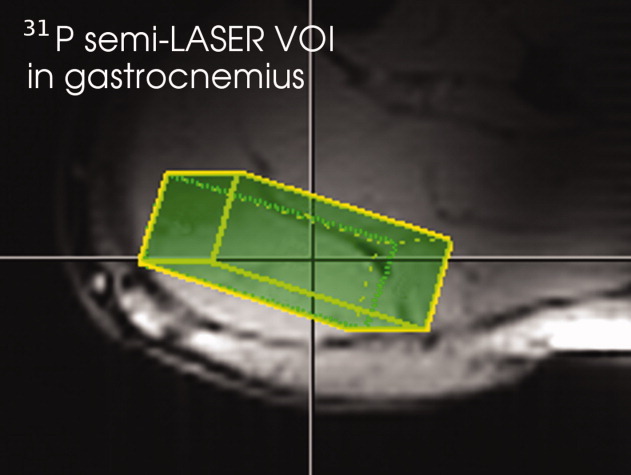
Position of the VOI in gastrocnemius muscle.

A double-oblique voxel, localized adopting the point resolved spectroscopy (PRESS) sequence with two pairs of adiabatic refocusing pulses [semi-LASER (15)], was placed in the subjects' gastrocnemius muscle (Fig. 1). The VOI was adjusted to the muscle's size and ranged from 23.8 to 47.7 cm^3^, with average dimensions of 4.2 × 1.7 × 5.3 cm^3^. The longest side was oriented along the leg (approximately H-F direction), and the shortest side was approximately orthogonal to the RF coil (A-P direction; Fig. 1). The VOI position was determined using scout image scans with three orthogonal slices and multislice gradient echo images (matrix size = 108 × 128, 14 slices, 7 mm slice thickness, field of view = 13 × 16 × 20 cm, echo time TE = 5 ms, TR = 0.4 s).

The muscle lobe selected with the localization scheme was gastrocnemius medialis (GM), a muscle predominantly consisting of fast-twitch fibers. The average (±SD) cross-sectional area (CSA) of GM was 15 ±4 cm^2^ or 19% of the total CSA of all muscles (82 ± 16 cm^2^) in the subjects' calves at the position selected for measurement. The VOI (average CSA of the voxel: 7.2 ± 1.7 cm^2^ or 49 ± 15% of GM's CSA) was placed inside this muscle, avoiding overlap to adjacent muscle groups. Because of the low contamination, which was verified in phantom experiments, we can conclude that the selected muscle is exclusively medial gastrocnemius.

To compare the semi-LASER acquisition during exercise and recovery with stimulated echo acquisition mode (STEAM) localization, a technique that has been used previously in 3 T studies (19, 20), one subject took part on two study days, for independent measurements with both localization schemes applied at 7 Tesla, another volunteer was examined using both localization strategies on the same day. STEAM measurements were performed with TE = 17 ms, the minimum TE achievable with sinc-shaped pulses (3.4 ms, BW = 2580 Hz). For semi-LASER, a classical, non-adiabatic, slice-selective, Shinnar-Le-Roux optimized 90° excitation pulse (2.6 ms, BW = 3400 Hz) was followed by two pairs of second-order hyperbolic secant (HS) adiabatic full passage pulses for refocusing (10 ms, BW = 2650 Hz), allowing a minimum TE = 53 ms. The TR was 8 s, comparable with previous measurements at 3 T (19), other measurement parameters were identical.

**Fig. 2:**
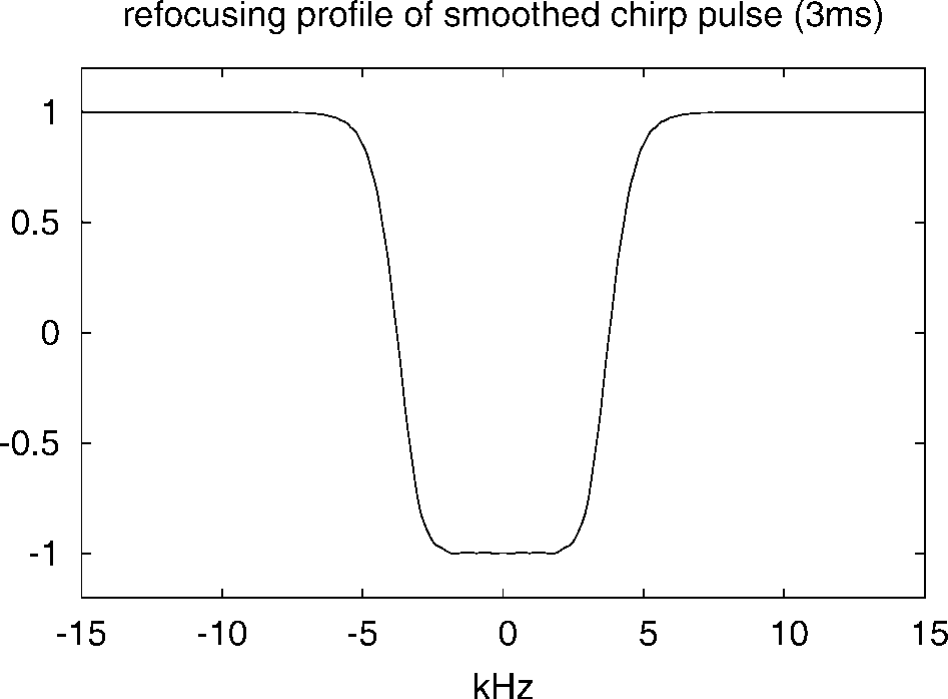
Simulated profile of the adiabatic chirp pulses used for slice-selective refocusing. Pulse duration was 3.0 ms, which resulted in a refocusing bandwidth of BW = 7600 Hz.

The semi-LASER sequence was then further optimized by shortening TR to 6 s to achieve high SNR per unit time for PCr and inorganic phosphate (Pi) with their *T*_1_ relaxation times of 4.0 ± 0.2 s and 6.3 ± 1.0 s, respectively, at 7 T (21). To reduce the TE, two pairs of smoothed chirp pulses with 3 ms duration and resulting BW = 7600 Hz were used for slice-selective adiabatic refocusing, resulting in minimum TE = 23 ms. The simulated profile of the chirp pulses is shown in Fig. 2. This simulation was successfully verified in a phantom measurement by slice-selective refocusing and readout of the signal under a frequency-encoding gradient.

The RF pulse durations were adapted depending on the required RF power, with the maximum transmitter voltage applicable to the surface coil as the limiting factor. This slightly increased the minimum achievable TE to 24 ms in two subjects (with 3.4 ms, BW = 6700 Hz refocusing pulses) and 26 ms (3.8 ms, BW = 6000 Hz) in one subject, for the given position and geometry of the VOI in the gastrocnemius muscle.

In all ^31^P MRS experiments, the acquisition bandwidth was 5000 Hz with 2048 complex data points, each acquisition vector was stored separately, without averaging. Quantification of spectra was done in jMRUI (22), using the AMARES (23) time domain fit routine. Gaussian line shapes were found to result in a better match to line shapes of the localized in vivo data than Lorentzian shapes. Widths of the lines (fitted without any apodization applied) are given using the relation between the damping parameter Γ/π of a gaussian line (24) (which is the numerical result of jMRUI) and full width at half maximum according to full width at half maximum 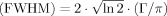. Cytosolic pH was calculated from the chemical shift difference of Pi and PCr in jMRUI, using *pK* = 6.75, δ_HA_ = 3.27, and δ_A_ = 5.63.

### Tests of the Localization Performance

**Fig. 3:**
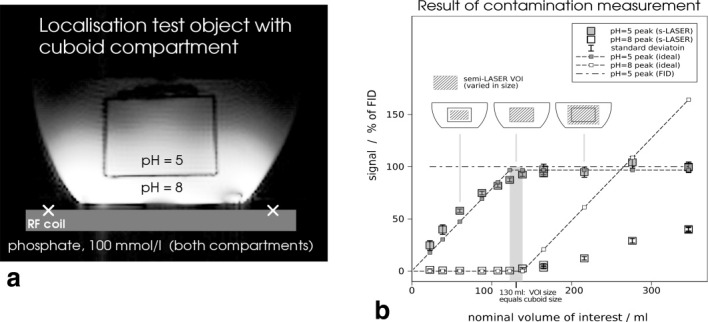
**a**: Scout image of the two compartment test object for measuring localization performance in singe-voxel MRS (white crosses: coil position). **b**: Contamination and selection efficiency were measured by acquiring spectra with the semi-LASER localization sequence under variation of voxel size. Gray squares represent the measured areas of the peak attributed to the inner compartment (pH = 5, the inner signal), and white squares show contributions from the outer substance (pH = 8, contamination).

The contamination of signals of the selected voxel with signals originating from outside the nominal VOI was quantified using a two-compartment test object filled with equally concentrated phosphate buffer solutions (*C* = 100 mmol/l) and a pH of 5 for the inner and pH of 8 for the outer compartment. Solutions contained saline in physiologic concentration for coil loading and were doped with gadolinium to shorten *T*_1_ relaxation times. The chemical shift difference between compartments was 2.5 ppm, resulting in a unambiguous separation of the resonances, exhibiting a FWHM of 6 Hz and 35 Hz, respectively. The inner compartment was formed by a cuboid acrylic glass box (5 × 3.5 × 7 cm^3^) with 1.2 mm wall thickness (Fig. 3a). Spectra were acquired to estimate the contamination of localized spectra by contributions from the outer moiety. Measurements were performed under fully relaxed conditions (TR = 30 s, i.e., > 7 · *T*_1_ of the phantom solutions), and corrections for different *T*_2_ relaxation of the inner and outer compartment were taken into account for calculating contamination.

The gray bar in Fig. 3b represents the volume of the acrylic cuboid walls, and the horizontal dash-dotted line corresponds to the signal from the inner moiety, measured with a (non-localized) pulse-acquire scheme, which demonstrates the efficiency of the semi-LASER sequence (under fully relaxed conditions).

### SNR Comparisons

For directly comparing the SNR of the 7 T MR system with 3 T systems, a test object consisting of 100 mmol/L phosphate in a 2 liter bottle was measured in two Siemens MR systems, using similar coils (loop coil, Ø ∼ 10 cm, both manufactured by Rapid Biomedical). The RF pulses were adjusted to achieve maximum signal under fully relaxed conditions. Evaluation of SNR was performed by dividing the phosphate peak's amplitude by the standard deviation of noise in a flat region (width: 1/8 of total BW) around 10 ppm up-field from the center frequency.

To give a more detailed analysis of the SNR gain under in vivo conditions, two comparisons were made. First, fully relaxed pulse-acquire spectra from calf muscle of the subjects measured at 7 T were compared with data acquired from eight subjects measured in a similar experiment during a study on a Siemens Trio (3 Tesla) scanner (25), using the RF coil mentioned above. The experiments at 3 T were repeated on several days, and a total of 26 measurements were analyzed. FWHM of the non-localized PCr signal in vivo was 7 ± 2 Hz at 3 T and 16 ± 7 Hz at 7 T.

Second, we compared the SNR of STEAM localized measurements at 7 T with data acquired with this sequence on a Bruker Medspec 3 Tesla system (26), using a ^31^P/^1^H surface coil (*d* = 10 cm, manufactured by Bruker). In all cases, the VOI was located in the medial gastrocnemius muscle. Data of seven subjects with a VOI size of 31 ± 2.4 cm^3^ and TE = 7.5 ms (3 T) were compared with data from two subjects measured at 7 T, with a VOI of 31 ± 1.6 cm^3^, TE = 13 ms, and 17 ms, respectively. Spectra with an equal number of averages (8 or 4) were compared.

### Exercise Protocol

After 2 min of rest (base line measurements), aerobic plantar flexion exercise was performed for 5 min, which induced significant PCr depletion and intracellular pH change (see Results section). Subsequently, acquisition of spectra continued during recovery for 7 min after the end of exercise. PCr was quantified from single acquisitions, while spectra were accumulated to improve SNR for Pi quantification and subsequent pH calculation during rest and recovery, when (resting) Pi concentration was low and may also be assumed to be constant in baseline conditions and the late recovery phase. PCr amplitudes of spectra acquired during recovery were fitted to an exponential function. Fitted parameters were PCr recovery rate constant *k*, given in s^−1^, and end-exercise PCr depletion *d*, given as % fraction of resting PCr concentration, *m*, according to the function *f*(*t*) = *m* · [1 − *d* · exp(−*tk*)]. In the Results section, the rate of PCr recovery is given as half time *t*_1/2_ (in seconds), which is reciprocally proportional to the rate constant according to *t*_1/2_ = ln(2) · *k*^−1^.

## RESULTS

Tests of the Localization Performance. The results of contamination and selection efficiency measurements obtained by varying the nominal voxel size in a two-compartment test object (Fig. 3a) are shown in Fig. 3b. Numerically, contamination was defined as a signal from the outer compartment divided by the sum of all signals from both compartments (26, 27), when nominally localizing precisely the inner compartment. Using the non-adiabatic 90° excitation pulse (for slice selection along *x* direction, parallel to the RF coil) and smoothed chirp pulses (for adiabatic refocusing), contamination was only 1.1 ± 0.5%. Selection efficiency, defined as the ratio of signal from the inner compartment using semi-LASER localization when compared with non-localized acquisition (the non-localized signal is represented by the dash-dotted line in Fig. 3), was 85 ± 1% when the nominal VOI coincided with the compartment and was 97 ± 3% when extending the VOI beyond the cuboid by 2 cm in each direction. With non-localized acquisition, signal from the outer compartment was 2.6 times larger than signal from the inner compartment, which shows the excellent suppression of undesired signal by the semi-LASER sequence.

### Signal-to-Noise Ratio

In a phantom, measured with a pulse-acquire scheme under fully relaxed conditions and with equal line widths, the SNR at 7 T was about two times higher than at 3 T (see Table 1), consistent with the findings published previously by our group (21).

**Table 1:** Comparisons of SNR from a Test Object and of In Vivo ^31^P MRS Muscle Data Obtained on Two 3 T Scanners and a 7 T Siemens Whole-Body System

SNR gain and FWHM changes (7 T vs. 3 T)	Phantom pulse-acquire fully relaxed Siemens Trio	In vivo pulse-acquire fully relaxed Siemens Trio	In vivo STEAM partial saturation Bruker Medspec 3T^a^
SNR (7 T/3 T)	1.8	2.0	2.6/3.5
FWHM (7 T/3 T)	0.9	2.3	1.7/1.1
SNR gain/rel. FWHM^b^	(1.7)	(4.7)	(4.5)/(3.9)

a Two SNR ratios given because results at 3 T were grouped into FWHM 5.5 or 3.5 Hz.

b Hypothetical SNR gain for equal linewidth.

In a comparison in vivo (Table 1), using a pulse-collect scheme under fully relaxed conditions on resting human calf muscle, we also found a 2-fold increase in SNR, despite a significant increase in line width at the higher field strength.

We also compared partially saturated localized ^31^P MR spectra of human medial gastrocnemius acquired with STEAM at 7 T (PCr FWHM = 6.0 ± 0.8 Hz) with data acquired with this sequence under comparable conditions at 3 T. As four of the data sets acquired at 3 T featured significantly narrower PCr lines (3.5 Hz) than the remaining datasets (5.5 Hz), two numbers are given for this SNR comparison (Table 1). This SNR comparison between 3 T and 7 T was carried out in frequency domain with the method described above and yielded nearly identical results when compared with the SNR obtained as the output of a time domain fit in jMRUI (i.e., factors of 2.4 and 3.3, respectively, again depending on line width).

Notably, the difference in field dependent SNR gain in vivo scales indirectly proportional to the increase in PCr line width, independent of the manufacturer of the MR systems. To eliminate the influence of line width, one can divide the 2-fold net SNR increase of 7T over 3T obtained on systems of the same manufacturer by the 2.3-fold line width increase for the non-localized measurements. This results in a similar SNR gain as the comparison of systems made by different manufacturers, showing SNR increases of 2.6 or 3.5. The result is a hypothetical SNR gain (i.e., numerically corrected for line width increase) of 4.4 ± 0.4.

Table 2 shows calculated signal losses due to relaxation and *J*-coupling for the semi-LASER localization sequence with the parameters used in this work. From this simulation, it is evident that quantification of uncoupled resonances with medium to long *T*_2_ are expected to suffer only from moderate losses (PCr: −10%, Pi: −19%) compared with acquisition strategies without spin echo. However, coupled resonances like adenosine triphosphate (ATP) will be decreased by 88% due to *T*_2_ decay and *J*-evolution.

**Table 2:** Calculated Losses Due To *T*_1_ and *T*_2_ Relaxation for PCr and Pi Measured with the Semi-LASER Sequence at Given TR = 6 s and TE = 23 ms

	PCr	Pi	γ-*ATP*
*T*_1_(s)	4.0	6.3	3.3
*T*_2_(ms)	217	109	29
*T*_1_ saturation	78%	61%	84%
*T*_2_ decay	90%	81%	45%
*J* evolution	–	–	27%

For γ-ATP [*J* = 18 Hz (26)] also the effect of *J* evolution is reported. Relaxation times were taken from Ref.^21^.

The SNR of the PCr peak at rest was acquired with semi-LASER 42 ± 4 across all subjects. SNR was measured in the frequency domain by measuring maximum peak amplitude in a spectrum measured with a single excitation after exponential apodization matched to the peak's FWHM and dividing by the standard deviation of noise in a flat region up-field from PCr. SNR was also quantified independently in four spectra acquired consecutively with TR = 6 s, during the resting phase (excluding the first acquisition). Consistently, the time domain fit routine AMARES in jMRUI yielded an average SNR of 31 ± 6. To give a figure of the sensitivity of the localized acquisition, SNR was quantified separately in four partially saturated (TR = 6 s) pulse-acquire spectra in all subjects, resulting in SNR = 270 ± 70. To relate the SNRs of non-localized with localized measurements, which showed different line shapes, SNRs calculated without apodization were compared, which yielded a 4.5 times higher SNR for unlocalized measurements. The expected ratio is 3.8, based on the simplified assumptions that the VOI of a pulse-acquire scheme is a hemisphere with the same radius as the surface coil and *T*_2_ correction factors from Table 2.

A comparison of SNR of the PCr peak in spectra acquired from resting muscle of the same subject with 32 signal averages, using the semi-LASER sequence (TE = 24 ms) and STEAM (TE = 10 ms) with otherwise equal parameters, resulted in a 2.1-fold higher SNR using semi-LASER. This signal gain is in good agreement with the expectation because STEAM inherently loses 50% of the signal and that adiabatic pulses achieve more efficient refocusing with the inhomogeneous *B*_1_ field of a loop coil used for RF transmission.

### Dynamic Localized In Vivo Measurements

High SNR, narrow peaks, and a flat base line enabled quantification of spectra from single acquisitions.

The line width of the PCr peak fitted as Gaussian lines was only 8.1 ± 2.2 Hz at rest, 9.5 ± 3.4 Hz during exercise, and returned to 8.5 ± 2.0 Hz during recovery across all subjects. This line width is half than the FWHM measured in non-localized experiments at rest in the same subjects, which was found to be 16 ± 7 Hz. Tip angles of 90° for the excitation pulses and adiabatic conditions for refocusing could be achieved with the given sequence parameters while keeping well within SAR limits and below the maximum RF transmit voltage applicable to the surface coil. The VOI was placed in the gastrocnemius muscle, which was located at a distance of 3 cm above the plane of the coil.

**Fig. 4:**
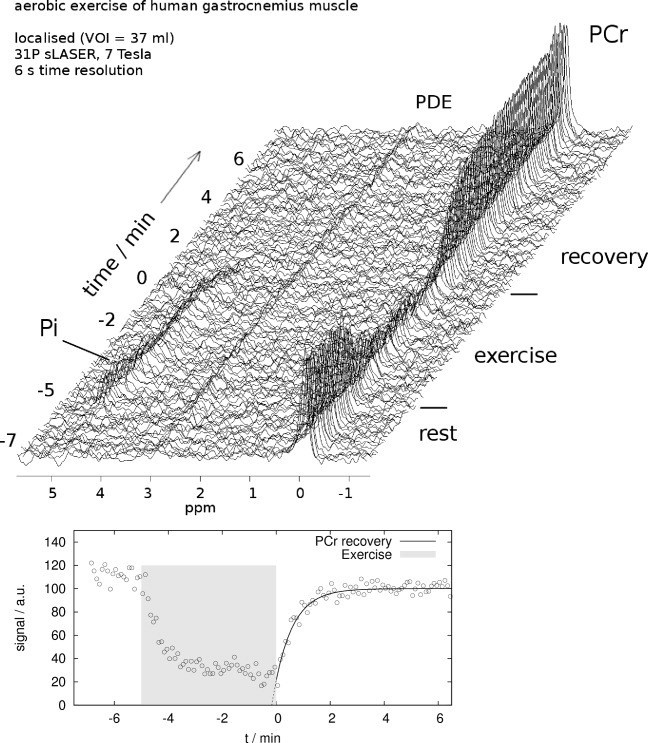
PCr recovery time course after aerobic plantar flexion exercise in a healthy subject measured with semi-LASER localized ^31^P MRS in a 7 T whole-body MR scanner (adiabatic refocusing: 3.0 ms smoothed chirp pulses, TE = 23 ms). Top: stack plot of spectra, no averaging, exponential line broadening 30 Hz (for display only). Bottom: fitted PCr time course.

It was possible to fit the PCr time courses to a model of exponential recovery using data acquired from a well-defined volume placed in a single exercising muscle (human GM), without the need to average spectra, which were acquired with a temporal resolution of 6 s (i.e., the TR of the sequence chosen based on expected optimum SNR per unit time). See Figure 4 for a stack plot of localized spectra, displayed without averaging, and fitted PCr exercise and recovery data.

**Fig. 5:**
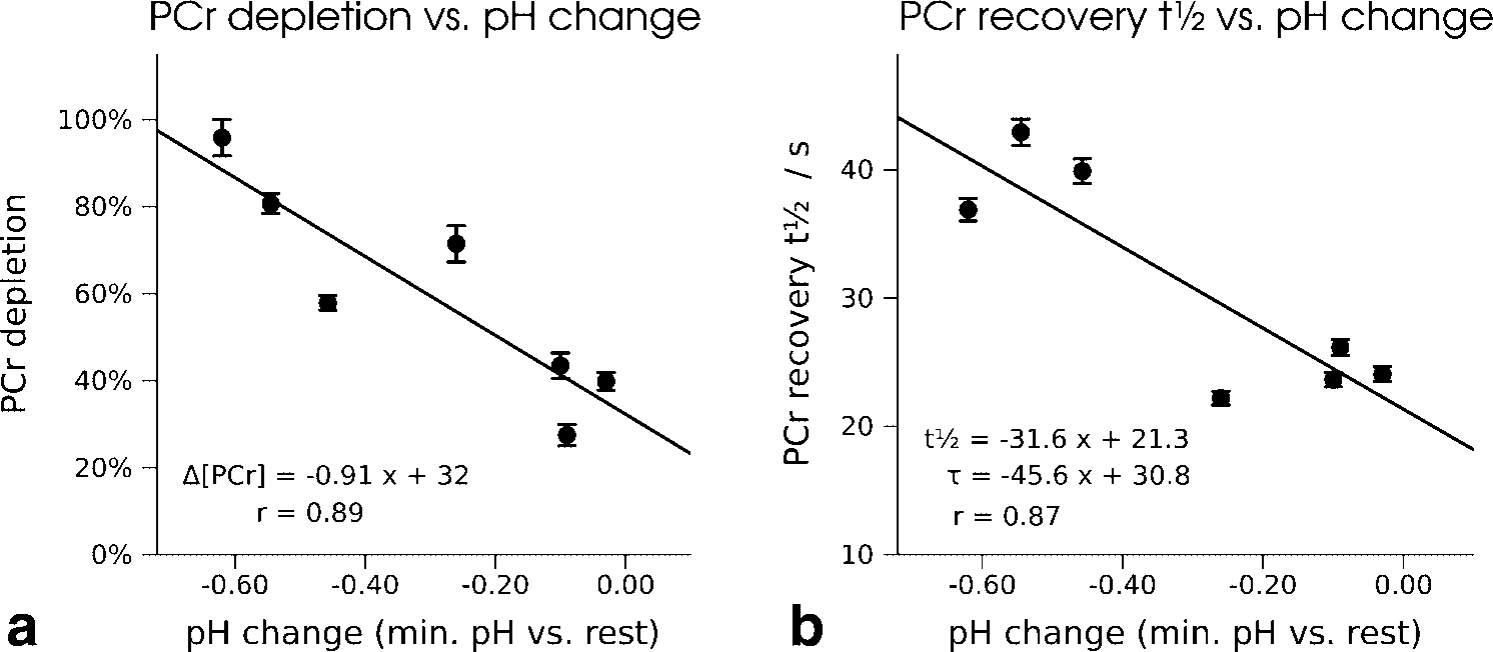
**a**: Correlation of pH change (given as the difference of resting pH and minimum pH reached after the end of exercise) and PCr depletion in percentage and **b**: correlation of pH and PCr recovery half time, measured in seven subjects in medial gastrocnemius muscle with ^31^P semi-LASER localization at 7 T. Parameters of the linear regression are given as half-time *t*_1/2_ and as characteristic time constant τ.

To follow pH changes, either four or eight consecutive spectra were averaged to enable robust fitting of Pi in resting conditions. The average pH in the subjects' gastrocnemius muscle was 7.04 ± 0.02 at rest and 6.87 ± 0.22 at the end of exercise, reaching a minimum of 6.74 ± 0.24 during early recovery. For characteristics of the PCr time courses measured with semi-LASER (TE = 23 ms, avg. VOI = 38 cm^3^), see table 3. Based on their maximum pH change and relative PCr depletion after 5 min of submaximal aerobic exercise, the subjects were assigned to one of the two groups: one group performed more strenuous exercise, and the other group was exercising to a lower extent. (Data acquired from a subject measured with TE = 53 ms were not included in this analysis.) Consistent with literature (28, 29), we observed a slower rate constant of PCr recovery after exercise, when pH decreases to lower levels at the end of exercise. Figure 5 shows correlations between minimum pH, reached on average 100 s after the end of exercise and relative PCr depletion and PCr recovery time.

**Table 3:** Mean values of maximum post exercise pH change, mean PCr depletion and PCr recovery rate constant (given as half time in seconds)

	|ΔpH_max_| range	|Δ[PCr]| range	*n*	|ΔpH_max_| Mean ± SD	|Δ[PCr]| Mean ± SD	PCr *t*_1/2_ Mean ± SD
Group 1	≤ 0.10	≤ 43%	3	0.07 ± 0.04	37 ± 8%	25 ± 1 s
Group 2	≥ 0.26	≥ 58%	4	0.47 ± 0.16	76 ± 16%	36 ± 9 s

Maximum intracellular pH changes after exercise and end exercise PCr depletions were used to assign data to one of two groups with distinct exercise levels.

The slope of the linear regression for the PCr recovery half-time *t*_1/2_ is - 31.6 s per unit pH change, which corresponds to a PCr recovery characteristic time τ correlation with 45.6 s/pH unit, in excellent agreement with the literature, reporting a proportionality constant of 46 s/pH unit (29) in a study of human gastrocnemius exercising at different levels.

**Fig. 6:**
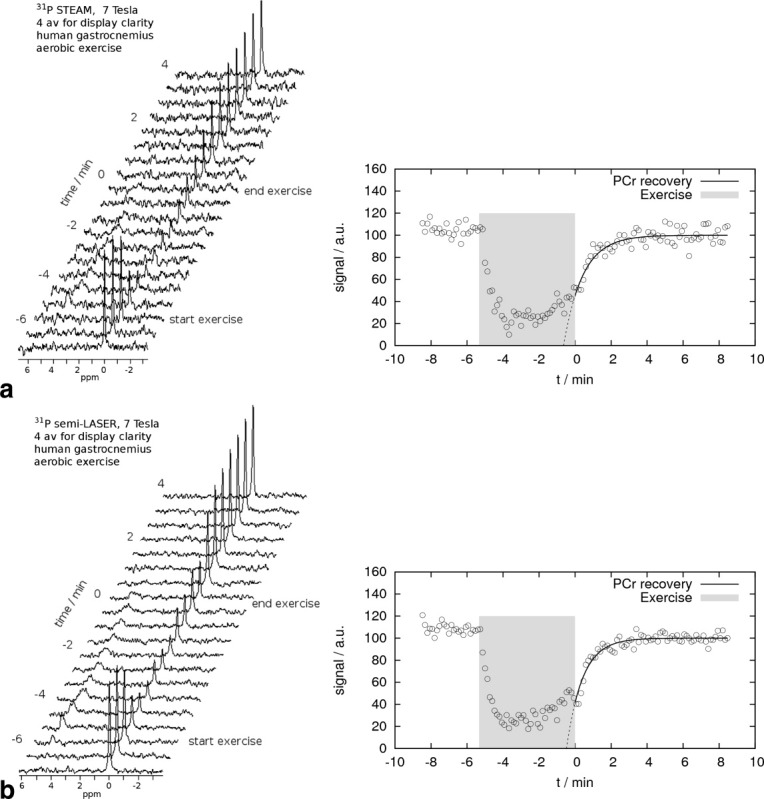
PCr recovery time course after aerobic plantar flexion exercise in a healthy subject measured with localized ^31^P MRS using a 7 T whole-body MR scanner. **a**: STEAM, TE = 17 ms. **b**: Semi-LASER with adiabatic hyperbolic secant refocusing pulses, TE = 53 ms. Left: stack plot of spectra (four averages for clarity of display only). Right: fitted PCr time course, no temporal averaging.

The increased SNR of semi-LASER when compared with STEAM is already obvious in the raw spectra (Fig. 6, left) as well as in the time course of fitted PCr data (Fig. 6, right), particularly during the period of PCr approaching its equilibrium value in the late phase of recovery. The PCr time course was measured with STEAM in two subjects showing 58 ± 5% PCr depletion and a recovery half-time of *t*_1/2_ = 34 ±14 s. After exercise, pH dropped from 7.05 ±0.01 to 6.9 ± 0.2, well within the range of data acquired with semi-LASER Localized.

## DISCUSSION AND CONCLUSION

It is demonstrated here that localized ^31^P MRS is capable of measuring metabolic changes with high temporal resolution in a single exercising muscle using a 7 T whole-body scanner. For localization, we used semi-LASER, a spectroscopic single-shot technique with 6 s TR, which allowed quantification of PCr from single acquisitions throughout the dynamic experiment. The SNR of PCr in GM muscle was 42 ± 4 under partial saturation without averaging. The FWHM of the PCr peak fitted as gaussian line was 8.1 ± 2.2 Hz. In previous work (19, 20), we showed that time-resolved ^31^P MRS is feasible at 3 T, using a similar dual-tuned (^1^H/^31^P) single-loop coil and STEAM localization. However, temporal resolution was reduced in comparison with pulse-acquire MRS experiments, which is the acquisition method commonly used for dynamic muscle MRS, because signal accumulation was necessary to increase SNR for reliable spectral quantification. At least two to four acquisitions were summed for PCr quantification in 3 T spectra of human gastrocnemius, depending on the lowest PCr value during exercise. Because of the signal gain at the higher *B*_0_ field, higher order shimming, and a decrease in spin-lattice relaxation times of most high energy phosphates, a strong gain in SNR was expected (21) and was successfully demonstrated to be achievable in a dynamic localized ^31^P MRS experiment applied to exercising muscle. The *B*_0_ field dependent line width increase by a factor of 1.1 to 1.7 for localized measurements is less pronounced than the 2.3-fold increase for pulse-acquire measurements.

The relatively long TE of semi-LASER applied with a surface coil at 7 T is a disadvantage for the detection of metabolites with short *T*_2_ or spin systems undergoing *J* modulation, such as ATP. Hence, ATP is detected with low SNR, making accumulation of a high number of spectra necessary (which is contradictionary to the benefit of a single-shot approach). Therefore, the focus of this work is on PCr and Pi quantification. At the minimum TE feasible in our setting, TE = 23 ms, the γ-ATP signal is decreased to 45% due to *T*_2_ relaxation, as given in Table 2 (in addition to *T*_1_ saturation to a level of 84% for TR = 6 s). The main source of signal loss is *J*-evolution, which decreases signal amplitude to 27%. This adds up to a total of only 10% of visible ATP signal, without considering the bandwidth-related chemical shift artifact. We verified the evolution of the ATP resonances under variation of TE with semi-LASER using a test object containing ATP in solution.

In the context of absolute quantification based on the the widely used assumption of ATP sustaining a constant concentration, the consequences are back-calculation factors with high numeric values. This potentially introduces large systematic errors in addition to the random error resulting from ATP quantification from low SNR data acquired by accumulating spectra (i.e., 64 averages would compensate for 90% of SNR loss induced by relaxation and *J*-modulation). Alternatively, localized MRS offers the possibility to use the phantom replacement technique using external reference standards. This quantification method can deliver highly accurate and reliable values for metabolite concentrations if relaxation is taken into account (30), without the necessity to rely on quantification of an internal metabolite assumed to sustain a constant concentration.

The bandwidth of the excitation pulse resulted in a chemical shift displacement artifact of 17% of the VOI for Pi and 8% for γ-ATP, with the carrier frequency set to the PCr resonance, while the smoothed chirp pulses used for refocusing resulted in a chemical shift displacement of 7% for Pi and 4% for γ-ATP. The A-P direction was considered particularly sensitive to the chemical shift artifact, as it is (a) orthogonal to the surface coil, and hence the direction of strongest *B*_1_ variations, and (b) anatomic variations are strongest along this axis, with subcutaneous fat, gastrocnemius, and soleus muscle following consecutively. Therefore, slice-selective refocusing was done in A-P direction, resulting in only 1.3 and 0.6 mm chemical shift displament for Pi and γ-ATP for the average voxel dimension (which was 17 mm in this direction). The respective shifts, given for Pi, along the directions parallel to the coil were 7 mm (excitation, voxel width = 42 mm) and 4 mm (second refocusing, length = 53 mm). For adiabatic refocusing, second-order hyperbolic secant and smoothed chirp pulses were also tested. While the pulse profile of hyperbolic secant pulses features steeper flanks, their time–bandwidth product is larger, and the minimum TE feasible with maximum *B*_1_ of the surface coil was 53 ms. With smoothed chirp pulses, excellent localization performance (approximately 1% contamination and 90% selection efficiency) was achieved with significantly shorter pulse durations and consequently higher excitation bandwidths and reduced chemical shift displacement artifact and TEs (minimum 23 ms).

In contrast to ^1^H spectroscopy, dynamic ^31^P MRS has often been used successfully without localization; however, because of several considerations, volume selection seems expedient also for non-proton MRS. Several studies using functional MRI of muscle [bold mfMRI (6, 31) and *T*_2_ imaging (5, 32)] have shown that muscle recruitment is not uniform. Thus, in an exercising limb, different muscles and distinct muscle groups contribute to contraction to a different extent, and depending on coil size and RF penetration depth, the metabolic state monitored by unlocalized ^31^P MRS represents an average across a heterogeneous ensemble of differently recruited compartments. Beyond that, the signal is weighted with a non-uniform *B*_1_ transmit and receive sensitivity profile if a surface coil is used. This heterogeneity of recruited muscles, depending on their relative size, position, and placement of the coil, contributing differently to the ^31^P MRS signal is a source of intersubject heterogeneity in MRS data (28). Localizing the acquired signal to a particular muscle should help eliminating this effect.

When compared with non-localized acquisition with the same surface coil, which in a rough estimation has a sensitive volume of approximately a half sphere with equal radius as the coil (*d* = 10.5 cm), spatial selection results in a decrease of the volume of tissue contributing to the spectral data acquisition by a factor of eight, from 300 cm^3^ to an average volume of 38 cm^3^. With a surface coil of this size, other muscles contributing to non-localized MRS acquisitions are mainly lateral gastrocnemius, lateral soleus, and medial soleus. Musculus peroneus brevis and m. tibialis posterior and the anterior muscle groups are smaller and located at a distance of more than 5 cm from the coil, so that their contribution to non-localized MR spectra can also be neglected. As a consequence of the 8-fold smaller volume selected, lower SNR is expected for localized measurements (which additionally uses an TE and hence results in *T*_2_ decay). In our experiments, we observed only 4.5 times less SNR with localized spectroscopy when compared with free induction decay acquisitions in vivo, at rest, which despite the *T*_2_ losses with localization can be explained by a decrease of line width by a factor of 2 and a more homogeneous excitation of the VOI due to localization and adiabatic refocusing.

In the past few years, several approaches have been made to perform dynamic localized ^31^P NMR, including gated chemical shift imaging measurements (33) and selective imaging of PCr alone or of PCr and Pi (34, 35). Our single-voxel approach, as a spectroscopic single-shot method, has the benefit of relatively high time resolution, retains a long TR (low *T*_1_ saturation, which can be quantified from relaxation data), and can be used to specifically select a single working muscle. Temporal resolution and SNR is sufficient to follow the time course of PCr using a multipoint exponential fit, time-resolved spectroscopic Pi and hence pH quantification with a lower but yet comparable temporal resolution, and the potential absolute quantification. We used TR = 6 s as a compromise between the optimum for PCr and Pi, it could be reduced further, if the focus is on PCr only and/or PCr depletion is low.

When ^31^P MRS is used to acquire information complementary to a localizing method, e.g., muscle-fMRI (31, 36), ^1^H MRS (19), or biopsies, the currently presented localized method would ensure that quantities from different methods originate from about the same volume. Also ^31^P signal from focal lesions may be of interest.

Finally, pulse-acquire experiments using surface coils, particularly with non-adiabatic excitation pulse shapes, are accompanied by strong variations of flip angles. The excitation of an inscribed VOI of a muscle suffers from much smaller flip angle variations, and adiabatic pulses further contribute to homogeneous signal acquisition. This is particularly interesting given the complex situation of PCr, a molecule that undergoes chemical exchange with γ-ATP and Pi, and therefore its relaxation characteristics are reflected as an apparent *T*_1_ (37–39). In the presence of muscle stimulation and, consequently, varying PCr and Pi concentrations, this chemical exchange might have an influence on measured apparent PCr recovery kinetics.

## SUMMARY

In conclusion, ^31^P MRS at 7 T benefits from increased SNR by a factor of 2 in phantoms and in vivo under fully relaxed conditions in non-localized spectra when compared with 3 T. Because of shortened *T*_1_s and a smaller increase of line width for localized spectroscopy, SNR can be improved by a factor of 3 under realistic conditions in vivo when choosing appropriate TRs. Further, we showed that using semi-LASER localization with adiabatic refocusing can yield double SNR when compared with STEAM at the same field strength.

The high selection efficiency and low contamination of the presented method demonstrate that it is suitable to collect dynamic MRS data from a single selected muscle, while retaining the high temporal resolution of a single-shot sequence. This metabolic information, as it is specific to a clearly defined single exercising organ, may provide additional value to the unambiguous interpretation of dynamic muscle MRS data in studies of normal physiology as well as muscular diseases.
